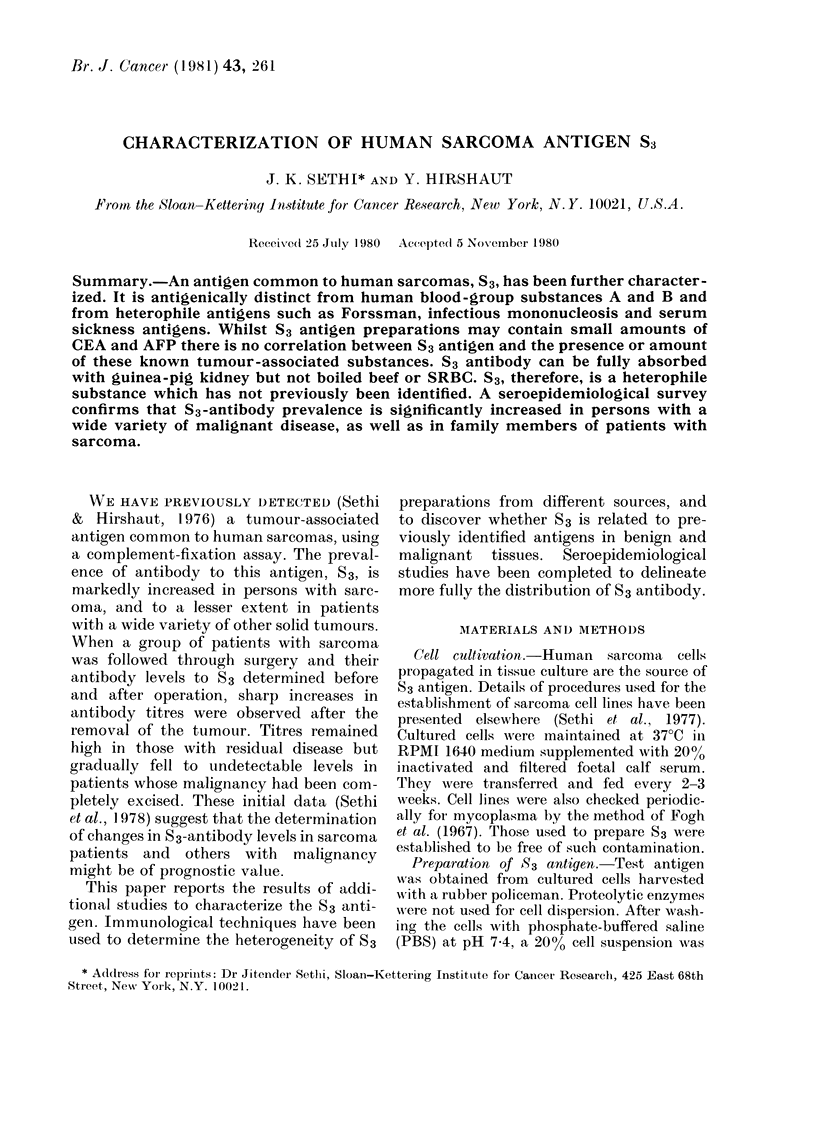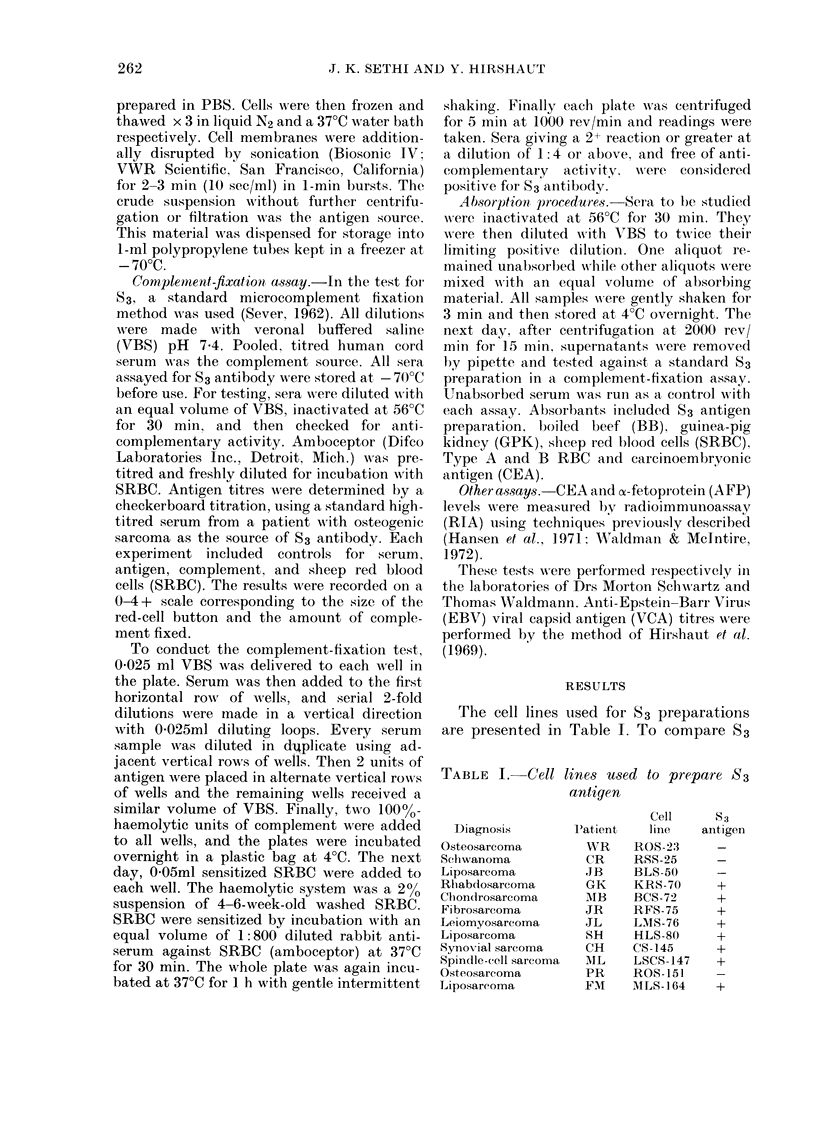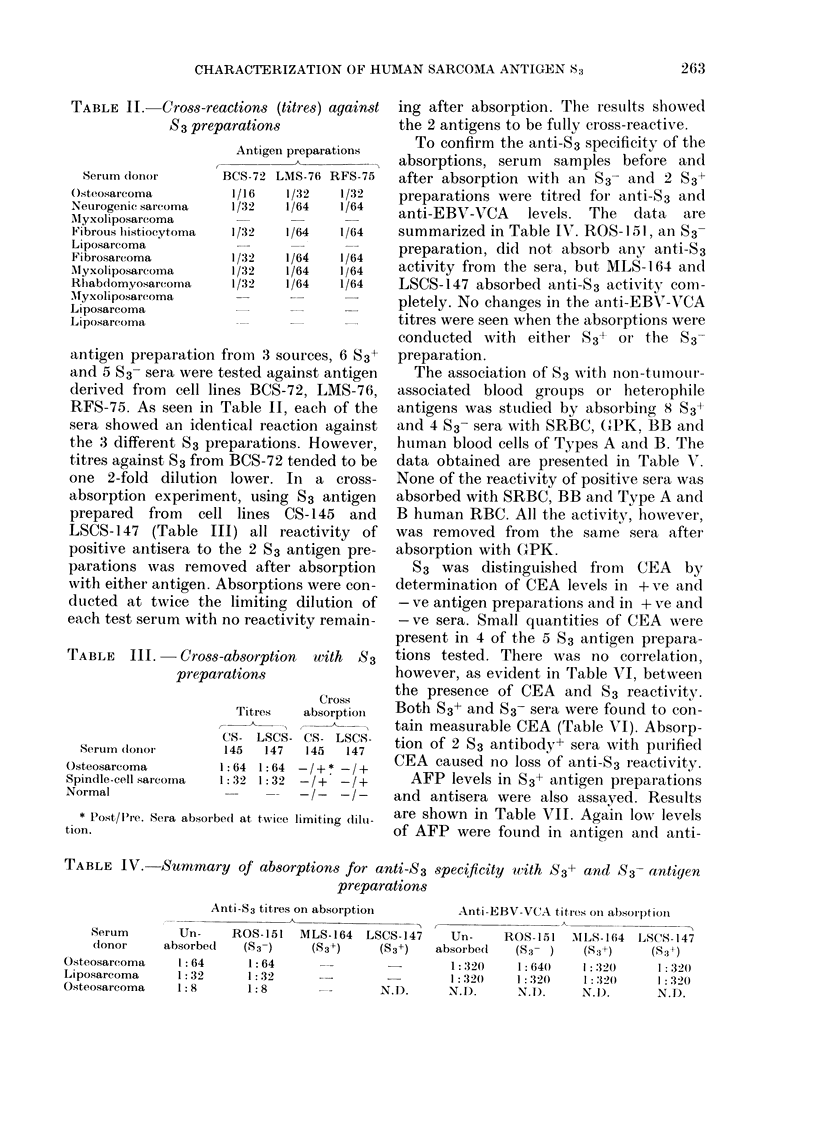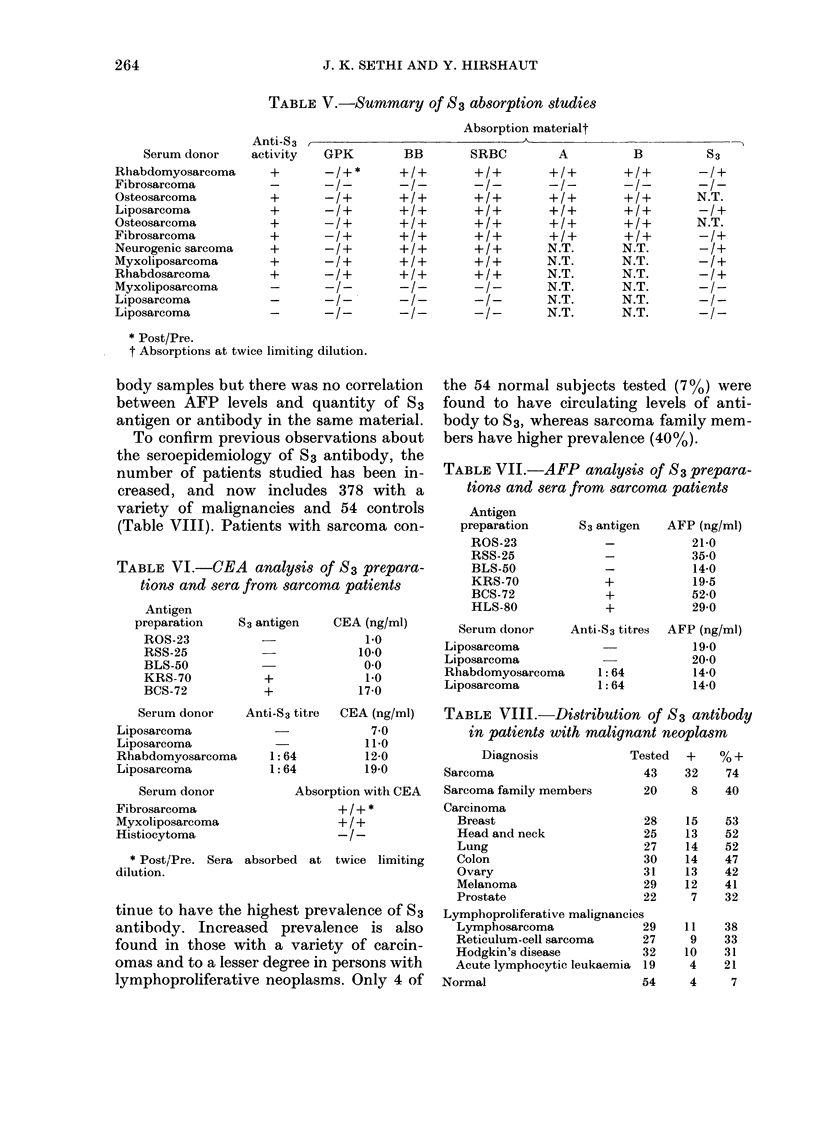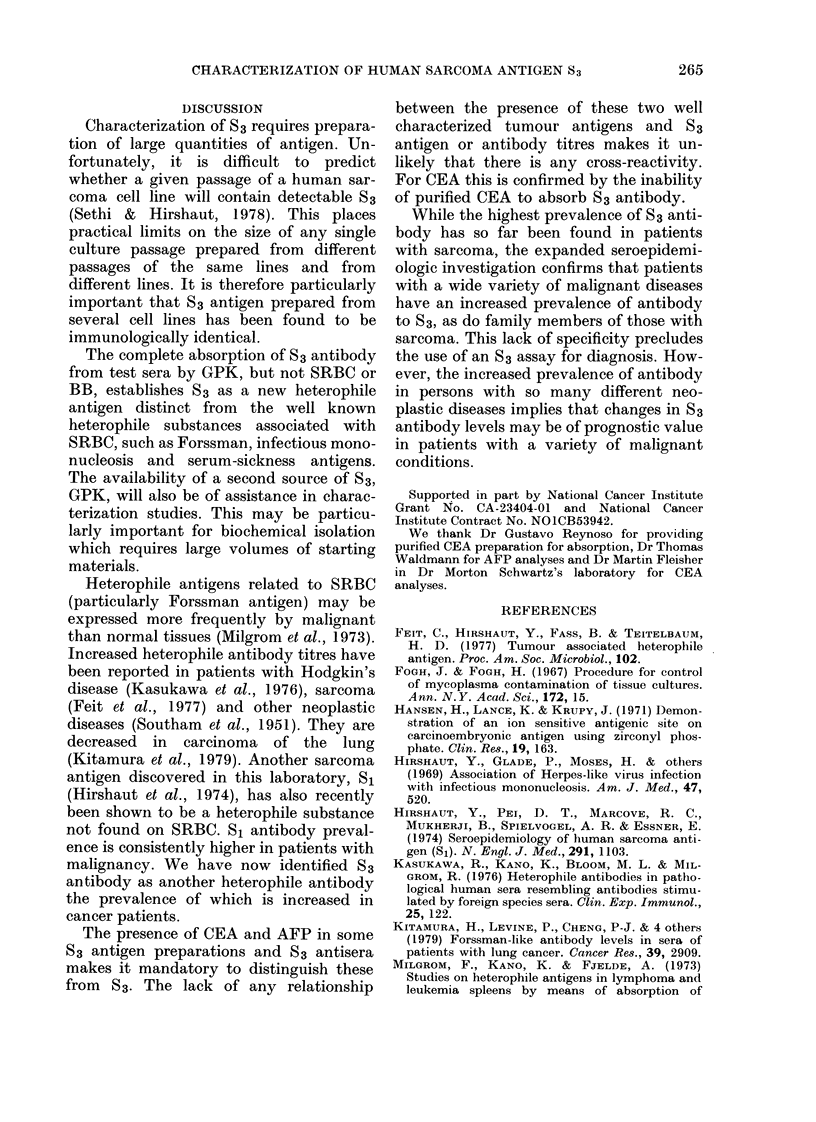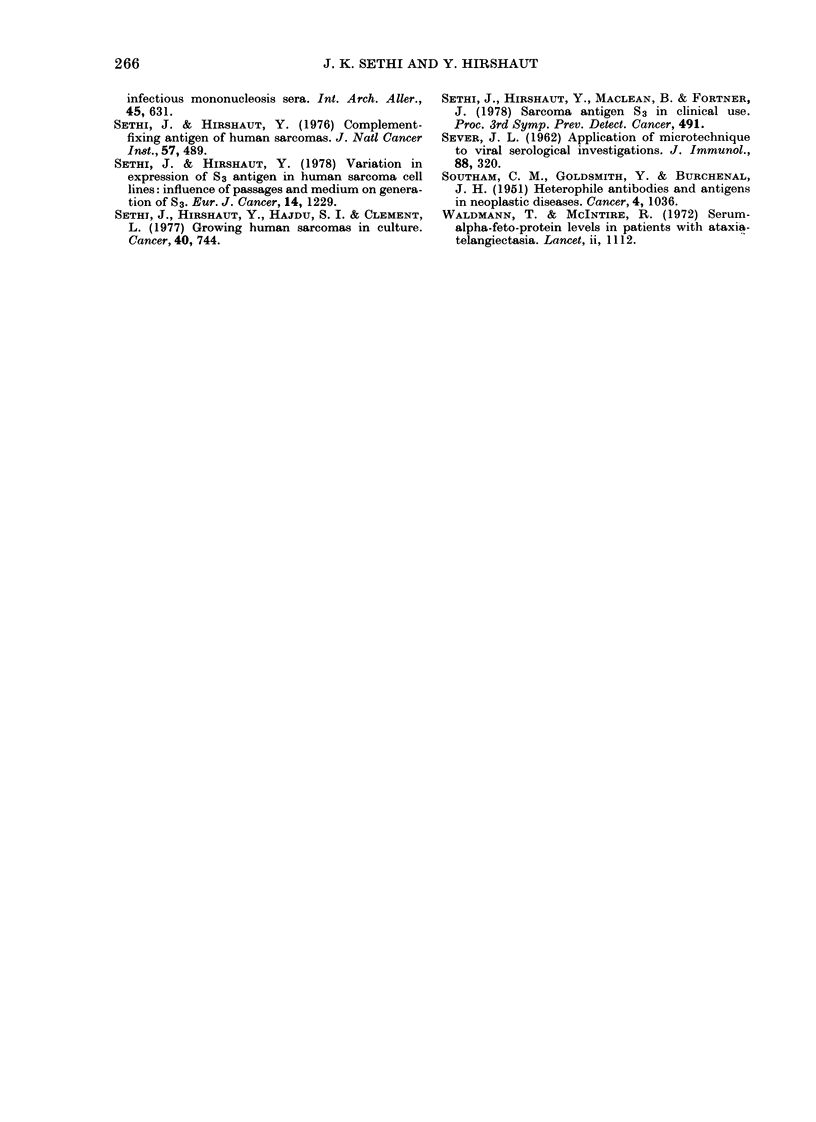# Characterization of human sarcoma antigen S3.

**DOI:** 10.1038/bjc.1981.43

**Published:** 1981-03

**Authors:** J. K. Sethi, Y. Hirshaut

## Abstract

An antigen common to human sarcomas, S3, has been further characterized. It is antigenically distinct from human blood-group substances A and B and from heterophile antigens such as Forssman, infectious mononucleosis and serum sickness antigens. Whilst S3 antigen preparations may contain small amounts of CEA and AFP there is no correlation between S3 antigen and the presence or amount of these known tumour-associated substances. S3 antibody can be fully absorbed with guinea-pig kidney but not boiled beef or SRBC. S3, therefore, is a heterophile substance which has not previously been identified. A seroepidemiological survey confirms that S3-antibody prevalence is significantly increased in persons with a wide variety of malignant disease, as well as in family members of patients with sarcoma.


*Br. J. Cancer* (1981) **43**, 261

### CHARACTERIZATION OF HUMAN SARCOMA ANTIGEN $S_3$

J. K. SETHI* AND Y. HIRSHAUT

*From the Sloan–Kettering Institute for Cancer Research, New York, N.Y.* 10021, *U.S.A.*

Received 25 July 1980 Accepted 5 November 1980

**Summary.—An antigen common to human sarcomas, $S_3$, has been further characterized. It is antigenically distinct from human blood-group substances A and B and from heterophile antigens such as Forssman, infectious mononucleosis and serum sickness antigens. Whilst $S_3$ antigen preparations may contain small amounts of CEA and AFP there is no correlation between $S_3$ antigen and the presence or amount of these known tumour-associated substances. $S_3$ antibody can be fully absorbed with guinea-pig kidney but not boiled beef or SRBC. $S_3$, therefore, is a heterophile substance which has not previously been identified. A seroepidemiological survey confirms that $S_3$-antibody prevalence is significantly increased in persons with a wide variety of malignant disease, as well as in family members of patients with sarcoma.**

WE HAVE PREVIOUSLY DETECTED (Sethi & Hirshaut, 1976) a tumour-associated antigen common to human sarcomas, using a complement-fixation assay. The prevalence of antibody to this antigen, $S_3$, is markedly increased in persons with sarcoma, and to a lesser extent in patients with a wide variety of other solid tumours. When a group of patients with sarcoma was followed through surgery and their antibody levels to $S_3$ determined before and after operation, sharp increases in antibody titres were observed after the removal of the tumour. Titres remained high in those with residual disease but gradually fell to undetectable levels in patients whose malignancy had been completely excised. These initial data (Sethi *et al.*, 1978) suggest that the determination of changes in $S_3$-antibody levels in sarcoma patients and others with malignancy might be of prognostic value.

This paper reports the results of additional studies to characterize the $S_3$ antigen. Immunological techniques have been used to determine the heterogeneity of $S_3$ preparations from different sources, and to discover whether $S_3$ is related to previously identified antigens in benign and malignant tissues. Seroepidemiological studies have been completed to delineate more fully the distribution of $S_3$ antibody.

### MATERIALS AND METHODS

*Cell cultivation.*—Human sarcoma cells propagated in tissue culture are the source of $S_3$ antigen. Details of procedures used for the establishment of sarcoma cell lines have been presented elsewhere (Sethi *et al.*, 1977). Cultured cells were maintained at 37°C in RPMI 1640 medium supplemented with 20% inactivated and filtered foetal calf serum. They were transferred and fed every 2–3 weeks. Cell lines were also checked periodically for mycoplasma by the method of Fogh *et al.* (1967). Those used to prepare $S_3$ were established to be free of such contamination.

*Preparation of $S_3$ antigen.*—Test antigen was obtained from cultured cells harvested with a rubber policeman. Proteolytic enzymes were not used for cell dispersion. After washing the cells with phosphate-buffered saline (PBS) at pH 7·4, a 20% cell suspension was

* Address for reprints: Dr Jitender Sethi, Sloan–Kettering Institute for Cancer Research, 425 East 68th Street, New York, N.Y. 10021.

prepared in PBS. Cells were then frozen and thawed $\times 3$ in liquid $N_2$ and a 37°C water bath respectively. Cell membranes were additionally disrupted by sonication (Biosonic IV; VWR Scientific, San Francisco, California) for 2–3 min (10 sec/ml) in 1-min bursts. The crude suspension without further centrifugation or filtration was the antigen source. This material was dispensed for storage into 1-ml polypropylene tubes kept in a freezer at −70°C.

*Complement-fixation assay.*—In the test for $S_3$, a standard microcomplement fixation method was used (Sever, 1962). All dilutions were made with veronal buffered saline (VBS) pH 7·4. Pooled, titred human cord serum was the complement source. All sera assayed for $S_3$ antibody were stored at −70°C before use. For testing, sera were diluted with an equal volume of VBS, inactivated at 56°C for 30 min, and then checked for anti-complementary activity. Amboceptor (Difco Laboratories Inc., Detroit, Mich.) was pre-titred and freshly diluted for incubation with SRBC. Antigen titres were determined by a checkerboard titration, using a standard high-titred serum from a patient with osteogenic sarcoma as the source of $S_3$ antibody. Each experiment included controls for serum, antigen, complement, and sheep red blood cells (SRBC). The results were recorded on a 0–4+ scale corresponding to the size of the red-cell button and the amount of complement fixed.

To conduct the complement-fixation test, 0·025 ml VBS was delivered to each well in the plate. Serum was then added to the first horizontal row of wells, and serial 2-fold dilutions were made in a vertical direction with 0·025ml diluting loops. Every serum sample was diluted in duplicate using adjacent vertical rows of wells. Then 2 units of antigen were placed in alternate vertical rows of wells and the remaining wells received a similar volume of VBS. Finally, two 100%-haemolytic units of complement were added to all wells, and the plates were incubated overnight in a plastic bag at 4°C. The next day, 0·05ml sensitized SRBC were added to each well. The haemolytic system was a 2% suspension of 4–6-week-old washed SRBC. SRBC were sensitized by incubation with an equal volume of 1:800 diluted rabbit anti-serum against SRBC (amboceptor) at 37°C for 30 min. The whole plate was again incubated at 37°C for 1 h with gentle intermittent shaking. Finally each plate was centrifuged for 5 min at 1000 rev/min and readings were taken. Sera giving a $2^+$ reaction or greater at a dilution of 1:4 or above, and free of anti-complementary activity, were considered positive for $S_3$ antibody.

*Absorption procedures.*—Sera to be studied were inactivated at 56°C for 30 min. They were then diluted with VBS to twice their limiting positive dilution. One aliquot remained unabsorbed while other aliquots were mixed with an equal volume of absorbing material. All samples were gently shaken for 3 min and then stored at 4°C overnight. The next day, after centrifugation at 2000 rev/min for 15 min, supernatants were removed by pipette and tested against a standard $S_3$ preparation in a complement-fixation assay. Unabsorbed serum was run as a control with each assay. Absorbants included $S_3$ antigen preparation, boiled beef (BB), guinea-pig kidney (GPK), sheep red blood cells (SRBC), Type A and B RBC and carcinoembryonic antigen (CEA).

*Other assays.*—CEA and α-fetoprotein (AFP) levels were measured by radioimmunoassay (RIA) using techniques previously described (Hansen *et al.*, 1971; Waldman & McIntire, 1972).

These tests were performed respectively in the laboratories of Drs Morton Schwartz and Thomas Waldmann. Anti-Epstein–Barr Virus (EBV) viral capsid antigen (VCA) titres were performed by the method of Hirshaut *et al.* (1969).

### RESULTS

The cell lines used for $S_3$ preparations are presented in Table I. To compare $S_3$

TABLE I.—*Cell lines used to prepare $S_3$ antigen*

| Diagnosis | Patient | Cell line | $S_3$ antigen |
|---|---|---|---|
| Osteosarcoma | WR | ROS-23 | − |
| Schwanoma | CR | RSS-25 | − |
| Liposarcoma | JB | BLS-50 | − |
| Rhabdosarcoma | GK | KRS-70 | + |
| Chondrosarcoma | MB | BCS-72 | + |
| Fibrosarcoma | JR | RFS-75 | + |
| Leiomyosarcoma | JL | LMS-76 | + |
| Liposarcoma | SH | HLS-80 | + |
| Synovial sarcoma | CH | CS-145 | + |
| Spindle-cell sarcoma | ML | LSCS-147 | + |
| Osteosarcoma | PR | ROS-151 | − |
| Liposarcoma | FM | MLS-164 | + |

TABLE II.—*Cross-reactions (titres) against $S_3$ preparations*

| Serum donor | Antigen preparations BCS-72 | LMS-76 | RFS-75 |
|---|---|---|---|
| Osteosarcoma | 1/16 | 1/32 | 1/32 |
| Neurogenic sarcoma | 1/32 | 1/64 | 1/64 |
| Myxoliposarcoma | — | — | — |
| Fibrous histiocytoma | 1/32 | 1/64 | 1/64 |
| Liposarcoma | — | — | — |
| Fibrosarcoma | 1/32 | 1/64 | 1/64 |
| Myxoliposarcoma | 1/32 | 1/64 | 1/64 |
| Rhabdomyosarcoma | 1/32 | 1/64 | 1/64 |
| Myxoliposarcoma | — | — | — |
| Liposarcoma | — | — | — |
| Liposarcoma | — | — | — |

antigen preparation from 3 sources, 6 $S_3^+$ and 5 $S_3^-$ sera were tested against antigen derived from cell lines BCS-72, LMS-76, RFS-75. As seen in Table II, each of the sera showed an identical reaction against the 3 different $S_3$ preparations. However, titres against $S_3$ from BCS-72 tended to be one 2-fold dilution lower. In a cross-absorption experiment, using $S_3$ antigen prepared from cell lines CS-145 and LSCS-147 (Table III) all reactivity of positive antisera to the 2 $S_3$ antigen preparations was removed after absorption with either antigen. Absorptions were conducted at twice the limiting dilution of each test serum with no reactivity remaining after absorption. The results showed the 2 antigens to be fully cross-reactive.

TABLE III.—*Cross-absorption with $S_3$ preparations*

| Serum donor | Titres CS-145 | LSCS-147 | Cross absorption CS-145 | LSCS-147 |
|---|---|---|---|---|
| Osteosarcoma | 1:64 | 1:64 | –/+* | –/+ |
| Spindle-cell sarcoma | 1:32 | 1:32 | –/+ | –/+ |
| Normal | — | — | –/– | –/– |

* Post/Pre. Sera absorbed at twice limiting dilution.

To confirm the anti-$S_3$ specificity of the absorptions, serum samples before and after absorption with an $S_3^-$ and 2 $S_3^+$ preparations were titred for anti-$S_3$ and anti-EBV-VCA levels. The data are summarized in Table IV. ROS-151, an $S_3^-$ preparation, did not absorb any anti-$S_3$ activity from the sera, but MLS-164 and LSCS-147 absorbed anti-$S_3$ activity completely. No changes in the anti-EBV-VCA titres were seen when the absorptions were conducted with either $S_3^+$ or the $S_3^-$ preparation.

The association of $S_3$ with non-tumour-associated blood groups or heterophile antigens was studied by absorbing 8 $S_3^+$ and 4 $S_3^-$ sera with SRBC, GPK, BB and human blood cells of Types A and B. The data obtained are presented in Table V. None of the reactivity of positive sera was absorbed with SRBC, BB and Type A and B human RBC. All the activity, however, was removed from the same sera after absorption with GPK.

$S_3$ was distinguished from CEA by determination of CEA levels in +ve and –ve antigen preparations and in +ve and –ve sera. Small quantities of CEA were present in 4 of the 5 $S_3$ antigen preparations tested. There was no correlation, however, as evident in Table VI, between the presence of CEA and $S_3$ reactivity. Both $S_3^+$ and $S_3^-$ sera were found to contain measurable CEA (Table VI). Absorption of 2 $S_3$ antibody$^+$ sera with purified CEA caused no loss of anti-$S_3$ reactivity.

AFP levels in $S_3^+$ antigen preparations and antisera were also assayed. Results are shown in Table VII. Again low levels of AFP were found in antigen and anti-

TABLE IV.—*Summary of absorptions for anti-$S_3$ specificity with $S_3^+$ and $S_3^-$ antigen preparations*

| Serum donor | Anti-$S_3$ titres on absorption: Un-absorbed | ROS-151 ($S_3^-$) | MLS-164 ($S_3^+$) | LSCS-147 ($S_3^+$) | Anti-EBV-VCA titres on absorption: Un-absorbed | ROS-151 ($S_3^-$) | MLS-164 ($S_3^+$) | LSCS-147 ($S_3^+$) |
|---|---|---|---|---|---|---|---|---|
| Osteosarcoma | 1:64 | 1:64 | — | — | 1:320 | 1:640 | 1:320 | 1:320 |
| Liposarcoma | 1:32 | 1:32 | — | — | 1:320 | 1:320 | 1:320 | 1:320 |
| Osteosarcoma | 1:8 | 1:8 | — | N.D. | N.D. | N.D. | N.D. | N.D. |

TABLE V.—*Summary of $S_3$ absorption studies*

| Serum donor | Anti-$S_3$ activity | Absorption material† GPK | BB | SRBC | A | B | $S_3$ |
|---|---|---|---|---|---|---|---|
| Rhabdomyosarcoma | + | −/+* | +/+ | +/+ | +/+ | +/+ | −/+ |
| Fibrosarcoma | − | −/− | −/− | −/− | −/− | −/− | −/− |
| Osteosarcoma | + | −/+ | +/+ | +/+ | +/+ | +/+ | N.T. |
| Liposarcoma | + | −/+ | +/+ | +/+ | +/+ | +/+ | −/+ |
| Osteosarcoma | + | −/+ | +/+ | +/+ | +/+ | +/+ | N.T. |
| Fibrosarcoma | + | −/+ | +/+ | +/+ | +/+ | +/+ | −/+ |
| Neurogenic sarcoma | + | −/+ | +/+ | +/+ | N.T. | N.T. | −/+ |
| Myxoliposarcoma | + | −/+ | +/+ | +/+ | N.T. | N.T. | −/+ |
| Rhabdosarcoma | + | −/+ | +/+ | +/+ | N.T. | N.T. | −/+ |
| Myxoliposarcoma | − | −/− | −/− | −/− | N.T. | N.T. | −/− |
| Liposarcoma | − | −/− | −/− | −/− | N.T. | N.T. | −/− |
| Liposarcoma | − | −/− | −/− | −/− | N.T. | N.T. | −/− |

* Post/Pre.
† Absorptions at twice limiting dilution.

body samples but there was no correlation between AFP levels and quantity of $S_3$ antigen or antibody in the same material.

To confirm previous observations about the seroepidemiology of $S_3$ antibody, the number of patients studied has been increased, and now includes 378 with a variety of malignancies and 54 controls (Table VIII). Patients with sarcoma continue to have the highest prevalence of $S_3$ antibody. Increased prevalence is also found in those with a variety of carcinomas and to a lesser degree in persons with lymphoproliferative neoplasms. Only 4 of the 54 normal subjects tested (7%) were found to have circulating levels of antibody to $S_3$, whereas sarcoma family members have higher prevalence (40%).

TABLE VI.—*CEA analysis of $S_3$ preparations and sera from sarcoma patients*

| Antigen preparation | $S_3$ antigen | CEA (ng/ml) |
|---|---|---|
| ROS-23 | — | 1·0 |
| RSS-25 | — | 10·0 |
| BLS-50 | — | 0·0 |
| KRS-70 | + | 1·0 |
| BCS-72 | + | 17·0 |
| **Serum donor** | **Anti-$S_3$ titre** | **CEA (ng/ml)** |
| Liposarcoma | — | 7·0 |
| Liposarcoma | — | 11·0 |
| Rhabdomyosarcoma | 1:64 | 12·0 |
| Liposarcoma | 1:64 | 19·0 |
| **Serum donor** | | **Absorption with CEA** |
| Fibrosarcoma | | +/+* |
| Myxoliposarcoma | | +/+ |
| Histiocytoma | | −/− |

* Post/Pre. Sera absorbed at twice limiting dilution.

TABLE VII.—*AFP analysis of $S_3$ preparations and sera from sarcoma patients*

| Antigen preparation | $S_3$ antigen | AFP (ng/ml) |
|---|---|---|
| ROS-23 | − | 21·0 |
| RSS-25 | − | 35·0 |
| BLS-50 | − | 14·0 |
| KRS-70 | + | 19·5 |
| BCS-72 | + | 52·0 |
| HLS-80 | + | 29·0 |
| **Serum donor** | **Anti-$S_3$ titres** | **AFP (ng/ml)** |
| Liposarcoma | — | 19·0 |
| Liposarcoma | — | 20·0 |
| Rhabdomyosarcoma | 1:64 | 14·0 |
| Liposarcoma | 1:64 | 14·0 |

TABLE VIII.—*Distribution of $S_3$ antibody in patients with malignant neoplasm*

| Diagnosis | Tested | + | %+ |
|---|---|---|---|
| Sarcoma | 43 | 32 | 74 |
| Sarcoma family members | 20 | 8 | 40 |
| Carcinoma | | | |
| Breast | 28 | 15 | 53 |
| Head and neck | 25 | 13 | 52 |
| Lung | 27 | 14 | 52 |
| Colon | 30 | 14 | 47 |
| Ovary | 31 | 13 | 42 |
| Melanoma | 29 | 12 | 41 |
| Prostate | 22 | 7 | 32 |
| Lymphoproliferative malignancies | | | |
| Lymphosarcoma | 29 | 11 | 38 |
| Reticulum-cell sarcoma | 27 | 9 | 33 |
| Hodgkin's disease | 32 | 10 | 31 |
| Acute lymphocytic leukaemia | 19 | 4 | 21 |
| Normal | 54 | 4 | 7 |

### DISCUSSION

Characterization of $S_3$ requires preparation of large quantities of antigen. Unfortunately, it is difficult to predict whether a given passage of a human sarcoma cell line will contain detectable $S_3$ (Sethi & Hirshaut, 1978). This places practical limits on the size of any single culture passage prepared from different passages of the same lines and from different lines. It is therefore particularly important that $S_3$ antigen prepared from several cell lines has been found to be immunologically identical.

The complete absorption of $S_3$ antibody from test sera by GPK, but not SRBC or BB, establishes $S_3$ as a new heterophile antigen distinct from the well known heterophile substances associated with SRBC, such as Forssman, infectious mononucleosis and serum-sickness antigens. The availability of a second source of $S_3$, GPK, will also be of assistance in characterization studies. This may be particularly important for biochemical isolation which requires large volumes of starting materials.

Heterophile antigens related to SRBC (particularly Forssman antigen) may be expressed more frequently by malignant than normal tissues (Milgrom *et al.*, 1973). Increased heterophile antibody titres have been reported in patients with Hodgkin's disease (Kasukawa *et al.*, 1976), sarcoma (Feit *et al.*, 1977) and other neoplastic diseases (Southam *et al.*, 1951). They are decreased in carcinoma of the lung (Kitamura *et al.*, 1979). Another sarcoma antigen discovered in this laboratory, $S_1$ (Hirshaut *et al.*, 1974), has also recently been shown to be a heterophile substance not found on SRBC. $S_1$ antibody prevalence is consistently higher in patients with malignancy. We have now identified $S_3$ antibody as another heterophile antibody the prevalence of which is increased in cancer patients.

The presence of CEA and AFP in some $S_3$ antigen preparations and $S_3$ antisera makes it mandatory to distinguish these from $S_3$. The lack of any relationship between the presence of these two well characterized tumour antigens and $S_3$ antigen or antibody titres makes it unlikely that there is any cross-reactivity. For CEA this is confirmed by the inability of purified CEA to absorb $S_3$ antibody.

While the highest prevalence of $S_3$ antibody has so far been found in patients with sarcoma, the expanded seroepidemiologic investigation confirms that patients with a wide variety of malignant diseases have an increased prevalence of antibody to $S_3$, as do family members of those with sarcoma. This lack of specificity precludes the use of an $S_3$ assay for diagnosis. However, the increased prevalence of antibody in persons with so many different neoplastic diseases implies that changes in $S_3$ antibody levels may be of prognostic value in patients with a variety of malignant conditions.

Supported in part by National Cancer Institute Grant No. CA-23404-01 and National Cancer Institute Contract No. NO1CB53942.

We thank Dr Gustavo Reynoso for providing purified CEA preparation for absorption, Dr Thomas Waldmann for AFP analyses and Dr Martin Fleisher in Dr Morton Schwartz's laboratory for CEA analyses.

### REFERENCES

Feit, C., Hirshaut, Y., Fass, B. & Teitelbaum, H. D. (1977) Tumour associated heterophile antigen. *Proc. Am. Soc. Microbiol.*, **102**.

Fogh, J. & Fogh, H. (1967) Procedure for control of mycoplasma contamination of tissue cultures. *Ann. N.Y. Acad. Sci.*, **172**, 15.

Hansen, H., Lance, K. & Krupy, J. (1971) Demonstration of an ion sensitive antigenic site on carcinoembryonic antigen using zirconyl phosphate. *Clin. Res.*, **19**, 163.

Hirshaut, Y., Glade, P., Moses, H. & others (1969) Association of Herpes-like virus infection with infectious mononucleosis. *Am. J. Med.*, **47**, 520.

Hirshaut, Y., Pei, D. T., Marcove, R. C., Mukherji, B., Spielvogel, A. R. & Essner, E. (1974) Seroepidemiology of human sarcoma antigen ($S_1$). *N. Engl. J. Med.*, **291**, 1103.

Kasukawa, R., Kano, K., Bloom, M. L. & Milgrom, R. (1976) Heterophile antibodies in pathological human sera resembling antibodies stimulated by foreign species sera. *Clin. Exp. Immunol.*, **25**, 122.

Kitamura, H., Levine, P., Cheng, P-J. & 4 others (1979) Forssman-like antibody levels in sera of patients with lung cancer. *Cancer Res.*, **39**, 2909.

Milgrom, F., Kano, K. & Fjelde, A. (1973) Studies on heterophile antigens in lymphoma and leukemia spleens by means of absorption of infectious mononucleosis sera. *Int. Arch. Aller.*, **45**, 631.

SETHI, J. & HIRSHAUT, Y. (1976) Complement-fixing antigen of human sarcomas. *J. Natl Cancer Inst.*, **57**, 489.

SETHI, J. & HIRSHAUT, Y. (1978) Variation in expression of $S_3$ antigen in human sarcoma cell lines: influence of passages and medium on generation of $S_3$. *Eur. J. Cancer*, **14**, 1229.

SETHI, J., HIRSHAUT, Y., HAJDU, S. I. & CLEMENT, L. (1977) Growing human sarcomas in culture. *Cancer*, **40**, 744.

SETHI, J., HIRSHAUT, Y., MACLEAN, B. & FORTNER, J. (1978) Sarcoma antigen $S_3$ in clinical use. *Proc. 3rd Symp. Prev. Detect. Cancer*, **491**.

SEVER, J. L. (1962) Application of microtechnique to viral serological investigations. *J. Immunol.*, **88**, 320.

SOUTHAM, C. M., GOLDSMITH, Y. & BURCHENAL, J. H. (1951) Heterophile antibodies and antigens in neoplastic diseases. *Cancer*, **4**, 1036.

WALDMANN, T. & MCINTIRE, R. (1972) Serum-alpha-feto-protein levels in patients with ataxia-telangiectasia. *Lancet*, ii, 1112.